# Digitally translated Self-Administered Gerocognitive Examination (eSAGE): relationship with its validated paper version, neuropsychological evaluations, and clinical assessments

**DOI:** 10.1186/s13195-017-0269-3

**Published:** 2017-06-27

**Authors:** Douglas W. Scharre, Shu ing Chang, Haikady N. Nagaraja, Nicole E. Vrettos, Robert A. Bornstein

**Affiliations:** 1 Division of Cognitive Neurology, Department of Neurology, The Ohio State University Wexner Medical Center, 395 W. 12th Avenue, 7th Floor, Columbus, OH 43210 USA; 2 Division of Biostatistics, College of Public Health, The Ohio State University, Cunz Hall, 1841 Neil Avenue, Columbus, OH 43210 USA; 3 Neuropsychology Laboratory, Department of Psychiatry, The Ohio State University Wexner Medical Center, 1670 Upham Drive, Columbus, OH 43210 USA

**Keywords:** Cognitive assessment, Cognitive screening, Computerized testing, Self-administered test, Neuropsychological evaluation, Mild cognitive impairment, Early dementia

## Abstract

**Background:**

The original paper Self-Administered Gerocognitive Examination (SAGE) is a valid and reliable cognitive assessment tool used to identify individuals with mild cognitive impairment (MCI) or early dementia. We evaluated identical test questions in a digital format (eSAGE) made for tablet use with the goals of calibrating it against SAGE and establishing its association with other neuropsychological tests and clinical assessments of cognitive impairment.

**Methods:**

Subjects aged 50 and over who had taken SAGE were recruited from community and clinic settings. Subjects were randomly selected to participate in a clinical evaluation including neuropsychological evaluations. SAGE and eSAGE were administered using a crossover design. Subjects were identified as dementia, MCI, or normal based on standard clinical criteria. Associations were investigated using Spearman correlations, linear regression, and sensitivity and specificity measures.

**Results:**

Of the 426 subjects screened, 66 completed the evaluation. eSAGE score correlation to a battery of neuropsychological tests was 0.73 (*p* < 0.0001) with no significant difference between the paper and digital format. Spearman correlation of SAGE versus eSAGE was 0.88 (*p* < 0.0001), and they are related by the formula: eSAGE score = –1.05 + 0.99 × SAGE score. Since the slope is very close to 1 (*p* = 0.86) there is strong evidence that the scaling is identical between eSAGE and SAGE, with no scale bias. Overall, eSAGE scores are lower by an average of 1.21 and the decrease is statistically significant (*p* < 0.0001). For those subjects familiar with smartphones or tablets (one measure of digital proficiency), eSAGE scores are lower by an average of 0.83 points (*p* = 0.029). With a score 16 and higher being classified as normal, eSAGE had 90% specificity and 71% sensitivity in detecting those with cognitive impairment from normal subjects.

**Conclusions:**

Tablet-based eSAGE shows a strong association with the validated paper SAGE and a neuropsychological battery. It shows no scale bias compared to SAGE. Both have the advantage of self-administration, brevity, four interchangeable forms, and high sensitivity and specificity in detecting cognitive impairment from normal subjects. Their potential widespread availability will be a major factor in overcoming the many obstacles in identifying early cognitive changes.

**Trial registration:**

ClinicalTrials.gov, NCT02544074. Registered on 18 March 2015.

## Background

Dementia is a growing problem worldwide in both the numbers of afflicted individuals and the cost of their care. In the US alone, there is an estimated 5.4 million individuals with Alzheimer’s disease (AD) at a healthcare cost of $236 billion dollars [1]. Perhaps an additional 3–22% of those over 60 years of age may meet criteria for mild cognitive impairment (MCI) [2–5].

Evidence is mounting, especially for AD, that early treatments and potential new disease modifying therapies are most successful in the earliest stages [6, 7]. We unfortunately have a situation where individual patients with cognitive impairment, MCI, and early dementia are typically not diagnosed or identified in a timely fashion [8, 9] to take full advantage of these medications. Therefore, improving the early identification of cognitive impairment must be a priority. Conversations between primary care providers and their patients and families regarding cognitive changes need to start much earlier in the disease course. However, there are many barriers in achieving this goal. Many patients reside in regions with few resources and with limited dementia-knowledgeable healthcare providers, clinical staff, or advocates. Providers may not be sophisticated or experienced in knowing how to screen those with cognitive complaints, which tools to use, or how to administer them. More than 40% of patients with mild dementia are not detected and diagnosed by their healthcare provider [10–14]. In addition, many patients with MCI or early dementia have impaired insight [15] and do not seek early medical intervention, typically only presenting to their family doctor an average of 3–4 years after cognitive symptoms are noticed by others [9, 16, 17]. There are also some family members who explain away the patient’s symptoms, reluctant to accept that their cognitive changes are meaningful. Other barriers include issues of limited reimbursement by Medicare for brief cognitive screening evaluations [18]. Providers and health systems may also have decided that too much time or personnel resources are required to administer cognitive testing more routinely.

The use of easily administered, brief, reliable, validated, practical, and inexpensive screening tools is critical in overcoming the many obstacles in identifying early cognitive changes in individuals. Screening Americans for cognitive impairment at their Medicare Annual Wellness Visit has been encouraged [19] and may provide a baseline prior to potential future decline in their cognitive abilities. Every individual has different natural abilities and so will have different baseline scores on their cognitive testing. There are many excellent cognitive screening tests with good sensitivity and specificity that can differentiate demented subjects from normal individuals [20–35]. Often they are underutilized due to the demand for personnel time and resources needed to administer them [36]. Many have not been evaluated for efficacy for MCI detection or have shown insensitivity in differentiating normal aging from MCI [37–41]. Informant-based assessments [42–45] may be limited due to lack of a readily accessible informant. Simpler cognitive tests that measure one or two cognitive domains, such as animal fluency, list learning tests, or the Mini-Cog test [46], have been advocated and used in primary care settings as a cognitive screen to be followed by more sensitive tests if impairments are noted [47].

We developed the Self-Administered Gerocognitive Examination (SAGE), a valid and reliable, 22-point traditional pen and paper multidomain cognitive assessment tool to reduce the typical delay in identifying individuals with MCI or dementia (available for download at sagetest.osu.edu) [48]. Our 2010 paper [48] describes in detail the reliability and validity study of the SAGE test. It establishes inter-rater and test-retest reliability, and equivalence of the four different versions of the test. It also correlated well with other cognitive measures of the same construct. The SAGE test was shown to have high sensitivity and specificity in distinguishing between normal, MCI, and dementia groups. The self-administered feature with age and education norms and four equivalent interchangeable forms of SAGE allows it to be given in almost any setting [49]. It takes on the average 13 min to complete and 30–60 s for it to be scored. It is sensitive enough to distinguish between MCI and dementia conditions and has been compared with other commonly used office-based multidomain brief cognitive tests [48, 50].

In recent years, as more individuals gain access and become comfortable with the Internet, they are also accessing medical information online from wherever they live in the world. Online information provides critical knowledge to consumers who wish to improve their health. Many have a great worry about developing dementia and AD and so the time is right for new digital solutions to cognitive testing. Computerized cognitive testing has been available for years. Most are developed as stand-alone formal neuropsychological test batteries designed to aid diagnosis particularly for those patients with subtle or atypical patterns of cognitive impairment [51–53]. Some computerized tests have been shown to distinguish between MCI and normal subjects and demonstrate potential for use in a primary care setting with a completion times of 30 min or less [54–56]. Most do not have equivalent paper versions which would allow flexibility for individuals taking the test. Digital translations of brief paper cognitive assessments have been developed and require validation in their own right [57].

In the present study, to provide a practical digital solution to early cognitive detection, a digital version of the paper SAGE test (eSAGE) made for tablet use is evaluated. The questions used in SAGE and eSAGE are identical. We evaluated eSAGE by comparing it to the validated SAGE, Mini-Mental State Examination (MMSE), Montreal Cognitive Assessment (MoCA), and a battery of neuropsychological tests. We measured the ability of eSAGE to detect MCI and early dementia against standard clinical assessment and neuropsychological evaluation. Having a validated online cognitive screening test may be very helpful for individuals to identify their cognitive issues, to prompt physician evaluation earlier than normally occurs, and to provide reassessments of their cognitive status.

## Methods

### eSAGE test development and description

The original SAGE has been previously described in detail (available for download at sagetest.osu.edu) [48]. The digital version of SAGE (eSAGE; commercially known as BrainTest**®**) made for tablet use, consisting of the identical test questions of SAGE, was produced by BrainTest Inc. (https://braintest.com) through a license agreement with The Ohio State University. The self-administered test measures cognitive function in the domains of orientation (date: 4 points), language (picture naming: 2 points; and verbal fluency: 2 points), memory (delayed recall of a written instruction: 2 points), executive function (modified Trails B: 2 points; and problem solving task: 2 points), abstraction (determining similarities: 2 points), calculations (word problem calculations: 2 points), and visuospatial abilities (copying three-dimentional constructions: 2 points; and clock drawing: 2 points). Nonscored items include demographic information (birth date, educational achievement, ethnicity, and sex), and questions regarding the individual’s past history of strokes and head trauma, family history of cognitive impairment, and current symptoms of memory, balance, mood, personality changes, and impairments of activities of daily living (ADL). No training is required for the administration of the test and assistance is not allowed. Clocks and calendars were not allowed in the testing rooms. Answers need not be spelled correctly. There is no time limit to complete the test. The subjects used their fingers to draw or type to complete the eSAGE questions on the tablet. A stylus was not permitted. The eSAGE design allowed for the subjects to write on the tablet as a scratch pad to aid in the completion of the calculations section. The subjects had the ability to delete and retype, or return and alter any answer to any question at any time. For those visuospatial and executive functioning questions requiring them to draw with their finger, there was the ability to erase their entire answer and redraw or redo their answer. If more than one response was provided for a question, the best response was scored. Upon completion of the eSAGE the responses were automatically uploaded. The eSAGE program tracked the timing of the responses and the number of erasures for an individual question. It also recorded the subjects drawings in real time so they could be played back later during scoring. Scoring was initially performed by an individual trained on the scoring instructions and verified by a second trained person. Any differences between scores were adjudicated and an agreement between the scorers reached. Detailed scoring instructions are the same for both SAGE and eSAGE and are available at sagetest.osu.edu.

### Study aims and approvals

The aim of the study was to compare and correlate the digital, tablet-based eSAGE with its validated paper SAGE and with the MMSE, MoCA, and a battery of neuropsychological tests. We also measured the ability of eSAGE to detect MCI and early dementia against standard clinical assessment and neuropsychological evaluation. This investigational study met institutional requirements for conduct of human subjects research and was registered on ClinicalTrials.gov (Identifier: NCT02544074). The Ohio State University’s Biomedical Sciences Human Subject Institutional Review Board approved it. Voluntary written informed consent was obtained from the subjects or their legally authorized representatives (when applicable) and assent was obtained from the subjects who were determined to not have the capacity to provide consent.

### Study participants

**Fig. 1:**
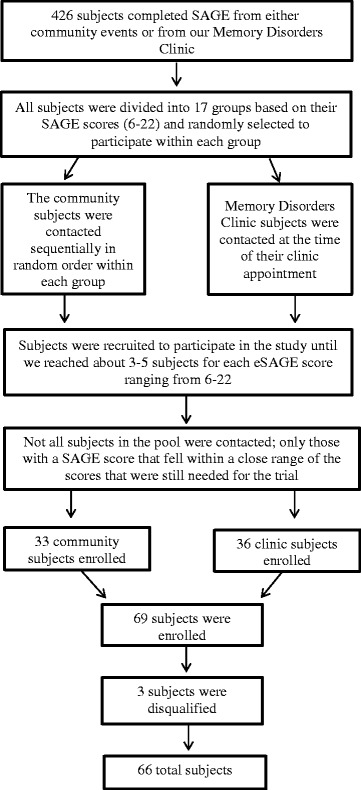
Participant flowchart. *eSAGE* Electronic Self-Administered Gerocognitive Examination, *SAGE* Self-Administered Gerocognitive Examination

Males and females 50 years of age and over with sufficient vision and hearing (determined through conversations with the subject/study partner and review of the subject’s medical history) who had taken SAGE given to them at educational talks to lay public, independent and assisted living facilities, senior centers, free memory screens or at the Memory Disorders Clinic at The Ohio State University were potential participants. Subjects with an intellectual disability and/or a diagnosis by a physician of moderate to severe dementia were excluded from the trial. Subjects completed SAGE between March 2014 and July 2016. The subjects who completed SAGE and were anticipated to meet the inclusion/exclusion criteria were divided into 17 groups based upon their SAGE score. Each group was comprised of subjects with the same score. The scores ranged from 6 to 22. The SAGE test forms for each group were numbered in numerical order. The numbers were then entered into Microsoft Excel and were randomly sorted for each group. The subjects were contacted per the randomization order and were asked to participate in a 1-day clinical evaluation (Fig. 1). The trial took place between January 2015 and July 2016. As an optional component to the trial, subjects were asked to identify a person who knows them well and would be willing to be interviewed as their study partner. Informed consent was obtained from all of the study partners. It was not exclusionary if the subject did not have a study partner.

### Clinical evaluation and study design

During the 1-day clinical evaluation a neuropsychological battery was administered to the subjects. Each patient was provided a different version of the SAGE test to the one they took initially. The battery consisted of the following assessments and was administered in this order: SAGE or eSAGE [48] (predetermined; see below), MMSE [26], Wisconsin Card Sort Test (WCST) [58, 59], eSAGE or SAGE [48] (the version not performed initially), MoCA [33], Hopkins Verbal Learning Test (HVLT) [60, 61], Wechsler Adult Intelligence Scale III (WAIS III) block design subtest [62], Boston Naming Test [63], FAS verbal fluency test [64] and the WAIS III letter number sequencing subtest [62]. The WAIS III block design subtest, the Boston Naming test, and the FAS verbal fluency test were administered during the waiting period for the HVLT delayed recall subtest. The SAGE and eSAGE were self-administered; however, a test supervisor was present during the examination. The test supervisor did not provide any assistance to the subjects but was present to document observations during the session and to record the completion time of the SAGE (the timing for the eSAGE was done automatically through the eSAGE program). There was no time limit; however, the completion time was recorded to allow for comparisons between the SAGE and eSAGE. The test supervisors scored the SAGE and eSAGE. The supervisor for the SAGE and eSAGE was different to the independent rater who administered and scored the rest of the neuropsychological battery. The independent rater was blinded to the SAGE and eSAGE scores and to the rest of the clinical evaluation. There were two independent raters and two SAGE/eSAGE supervisors who were involved in the trial. Training for the independent raters was conducted prior to their involvement in the trial to prevent variations in measurement. Training for SAGE/eSAGE scoring was also conducted prior to the supervisor’s involvement in the trial.

All seven neuropsychological scores were standardized with a mean of 50 and a standard deviation of 10 based on published normative data for these tests [65, 66]. The standardized scores were recorded separately for perseverative errors of the WSCT, WAIS III letter-number, WAIS III block design, Boston Naming Test, the FAS fluency test, HVLT total recall learning score (the sum of the correctly recalled words on all three learning trials), and the HVLT retention score (the total number of words remembered with delayed recall). The HVLT total recall learning score and the HVLT retention score were defined as the two memory items. These seven standardized neuropsychological test items were also summed to give a total (7-item total) neuropsychological score. We used this exact combination of neuropsychological measures in our original SAGE paper [48] to be consistent with our previous work.

In addition to the neuropsychological battery, a physical and neurological examination (conducted by DWS, other study physicians, or study nurse practitioners) including vitals was performed and a detailed medical history including a list of current medications was obtained from all subjects by the study coordinator through conversations with the subject/study partner and review of the subject’s medical records. The participants were queried and their responses were recorded regarding their previous experience with tablets, computers, smartphones, and similar electronic devices. Subjects were determined to be digitally proficient if they had previous experience with either smartphones or tablets. Behavioral and functional measures including the Neuropsychiatric Inventory (NPI) [67] and the ADL [68, 69] were administered to the study partners. If a subject did not have a study partner the measures were administered to the participant instead.

The SAGE and the eSAGE were administered through a crossover design. Upon enrollment the subjects were assigned one of the four versions of the SAGE test (form 1, 2, 3, or 4). To reduce learning effect, the form that was used for the clinical evaluation was different from the form that was used for their original SAGE test. The researchers, to ensure that there were equal representations of the different versions, balanced the number of subjects who were assigned to each form. The forms were assigned to the subjects in ascending order, beginning with form 1, as they were enrolled in the trial. If a subject was assigned to a form that corresponded to their original SAGE form the next numerical version was assigned instead. The SAGE and eSAGE form was the same for an individual subject. The order of administration for the SAGE and eSAGE was randomized through stratified randomization. The stratification was based upon the SAGE form. The order (SAGE or eSAGE administered first) alternated for each form as the subjects were enrolled in the trial.

Subjects were grouped by two trained dementia specialists blinded to their eSAGE, SAGE, MMSE, and MoCA scores as either dementia, MCI, or normal based on standard clinical criteria and their neuropsychological testing. Any differences between diagnostic groupings were adjudicated and an agreement between the dementia specialists reached. To maintain consistency, the criteria we used were identical to the criteria used in our original SAGE publication [48]. Specifically, dementia subjects were defined as scoring greater than two standard deviations below the mean for at least one of the two memory items and greater than two standard deviations below the mean for at least one of the nonmemory items or greater than two standard deviations below the mean for the 7-item total score. All dementia subjects met the memory impairment criteria. The subjects must also have had significant functional decline based on the ADL scale. If they did not, they were classified as MCI. This definition of dementia is consistent with standard clinical criteria [70]. Therefore, based on standard clinical criteria including ADL information, the trained dementia specialist could override the more specific neuropsychological test requirements noted above. One subject who met dementia criteria based on neuropsychological testing was diagnosed as MCI based on functional and clinical assessments.

The MCI group was made up of subjects who did not meet the criteria for dementia above but had scores greater than 1.5 standard deviations below the mean for at least one of the seven items or greater than 1.5 standard deviations below the mean for the 7-item total score. The subject must have had normal or slight impairment in functional abilities based on the ADL scale. If they had significant functional decline, they were classified as dementia. This definition of MCI is consistent with standard clinical criteria [71]. Five subjects who met MCI criteria based on neuropsychological testing were diagnosed as dementia based on functional and clinical assessments.

Normal subjects were defined as those that did not fit the dementia or MCI criteria above and had normal functional abilities based on the ADL scale.

Primary outcome measures for the trial were the correlation between the subject’s eSAGE score compared to their summed seven neuropsychological test scores, and the correlation and agreement between the eSAGE score and their SAGE score. The secondary outcome measures were the correlations between eSAGE and the other cognitive measures and the ability of eSAGE to detect MCI and early dementia against standard clinical assessment and neuropsychological evaluation.

### Statistical analyses

Associations between various test scores were investigated using Spearman correlations and equality of correlations from dependent variables were tested using the T_2_ statistic given by Steiger [72]. Age- and education-adjusted neuropsychological test scores were used in these correlations. Concordance correlation coefficient [73] was used as the measure of agreement between SAGE and eSAGE scores. Comparison of SAGE and eSAGE scores and the examination of the effect of digital proficiency were preformed using *t* tests and linear regression analyses. Receiver operating characteristics (ROC) curves were obtained, and specificity and sensitivity were determined for various cut-off values of SAGE and eSAGE. Comparison of the median duration of tests was based on first test scores using Wilcoxon rank-sum test. All pairwise comparisons of group means were carried out using Tukey’s HSD (Honest Significant Difference) test. Sensitivity and specificity were compared using McNemar’s chi-square test. Area under the ROC curves (AUC) were compared using the methodology described in Hanley and McNeil [74, 75]. A *p* value below 0.05 was considered significant. JMP 11.0 (SAS Institute, Cary, NC, USA) software was used for all statistical analyses.

## Results

### Participant demographics and baseline clinical characteristics

There were 426 individuals who completed SAGE from either community events or from our Memory Disorders Clinic. Sixty-nine subjects were enrolled and 66 subjects were recruited, as described in detail above, who met all eligibility criteria and completed the full clinical evaluation (Fig. 1). Of the 66, 52% (34 subjects) had a study partner. Table 1 provides other demographic data and the baseline clinical characteristics of the subjects. The average subject was aged in their mid-70s, with some post-high school education. Fifteen percent were less than 70 years old and 20% were over 80 years old. Three percent had less than high school education and 79% reported more than high school education. Two-thirds were women, just over 10% were minorities and half had no experience with using either a tablet or a smartphone. MMSE and MoCA cognitive scores ranged from normal to mild dementia.

**Table 1:** Subject demographics and characteristics

Age (years)	75.2 ± 7.3 (54–92)
Education (years)	15.1 ± 2.7 (8–21)
Sex, female	67%
Race, Caucasian	89%
Experience with tablet or smartphone	53%
Baseline SAGE score	14.3 ± 3.9 (6–22)
Clinical evaluation SAGE score	15.5 ± 4.5 (4–22)
eSAGE score	14.3 ± 5.0 (2–22)
MMSE score	26.9 ± 2.6 (20–30)
MoCA score	20.7 ± 4.5 (8–29)
7-Item total neuropsychology battery score	287.2 ± 51.8 (174–403)

Values are shown as mean ± SD (range) or %

*eSAGE* electronic Self-Administered Gerocognitive Examination, *MMSE* Mini-Mental State Examination, *MoCA* Montreal Cognitive Assessment, *SAGE* Self-Administered Gerocognitive Examination, *SD* standard deviation

### Clinical measures

#### eSAGE comparisons to neuropsychological tests

Table 2 provides the Spearman rank correlations between the digital and paper SAGE testing and the 7-item total of a battery of neuropsychological tests. eSAGE correlation to our neuropsychological battery was 0.73, which is not significantly different from the correlation of the paper format with this battery (*p* = 0.227). Correlations of SAGE and eSAGE to MoCA and MMSE are also highly significant.

**Table 2:** eSAGE and SAGE Spearman rank correlations to other cognitive assessments

Variable	By variable	Correlation	*p* value for correlation comparisons*
SAGE	eSAGE	0.8824	
MMSE	eSAGE	0.6711	0.4751
MMSE	SAGE	0.6388	
MMSE	MoCA	0.6939	
MoCA	eSAGE	0.7577	0.5612
MoCA	SAGE	0.7349	
Neuropsychological battery	eSAGE	0.7292	0.2271
Neuropsychological battery	SAGE	0.6784	
Neuropsychological battery	MMSE	0.5869	0.0416
Neuropsychological battery	MoCA	0.7274	

*Using T_2_ from Steiger [72]

Neuropsychological battery correlation comparisons: eSAGE vs MMSE (*p* = 0.0452); SAGE vs MMSE (*p* = 0.2384)

*eSAGE* electronic Self-Administered Gerocognitive Examination, *MMSE* Mini-Mental State Examination; *MoCA* Montreal Cognitive Assessment; *SAGE* Self-Administered Gerocognitive Examination

#### eSAGE comparisons to SAGE

**Fig. 2:**
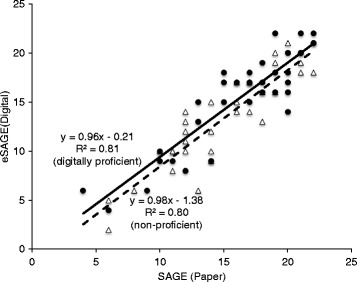
eSAGE scores compared to SAGE scores as a function of subject’s digital proficiency. For those subjects not familiar with either smartphones or tablets (one measure of digital proficiency), the scores (*open triangles*) are related by the formula: eSAGE score = –1.38 + 0.98 × SAGE score (*dashed line*); the coefficient of determination (*R* ^2^) is 0.80; the slope is not significantly different from 1 (*p* = 0.85), and the intercept is not significantly different from 0 (*p* = 0.33). For those subjects familiar with smartphones or tablets, the scores (*filled circles*) are related by the formula: eSAGE score = –0.21 + 0.96 × SAGE score (*solid line*); the coefficient of determination (*R* ^2^) is 0.81; the slope is not significantly different from 1 (*p* = 0.64); the intercept is not significantly different from 0 (*p* = 0.88). When combined, the scores are related by the formula: eSAGE score = –1.05 + 0.99 × SAGE score with *R* ^2^ = 0.81; the slope is not significantly different from 1 (*p* = 0.86), and the intercept is not significantly different from 0 (*p* = 0.28) (line not shown). *eSAGE* electronic Self-Administered Gerocognitive Examination, *SAGE* Self-Administered Gerocognitive Examination

Spearman correlation of SAGE versus eSAGE was 0.882 (*p* < 0.0001). eSAGE and SAGE are related by the formula: eSAGE score = –1.05 + 0.99 × SAGE score (Fig. 2). The slope of 0.99 is not significantly different from 1 (*p* = 0.86) showing strong evidence that the scaling is identical between eSAGE and SAGE. There is no evidence of a scale bias. The intercept is not significantly different from 0 (*p* = 0.28). Subjects’ eSAGE scores are lower by an average of 1.21 points from their SAGE scores (significant, *p* < 0.0001) whether they scored in the high, middle, or low ranges.

Of our subjects, 47% were judged to be not digitally proficient based on having no previous experience with either smartphones or tablets. A comparison between the two groups in terms of association between eSAGE and SAGE scores is given in Fig. 2. For those subjects not familiar with either smartphones or tablets (one measure of digital proficiency), the scores are related by the formula: eSAGE score = –1.38 + 0.98 × SAGE score; the coefficient of determination (*R* ^2^) is 0.80, the slope is not significantly different from 1 (*p* = 0.85), and the intercept is not significantly different from 0 (*p* = 0.33). For those subjects familiar with smartphones or tablets, the scores are related by the formula: eSAGE score = –0.21 + 0.96 × SAGE score; the coefficient of determination (*R* ^2^) is 0.81, the slope is not significantly different from 1 (*p* = 0.64), and the intercept is not significantly different from 0 (*p* = 0.88). For the digitally proficient group, eSAGE scores are lower than SAGE scores by an average of 0.83 points (*p* = 0.029 by paired *t* test; effect size = 0.17), and for the group that was not digitally proficient the average difference is 1.65 points (*p* = 0.0002 by paired t test; effect size = 0.36). The difference between these two average differences (0.82) was not statistically significant (*p* = 0.13). The concordance correlation coefficient [73], a common measure of agreement, between eSAGE and SAGE was 0.8687.

The median length of time it took to complete eSAGE was 17.5 min compared to 16 min for SAGE. There was no significant difference between the medians (*p* = 0.23 by Wilcoxon rank-sum test). Part of the total time recorded included the time to respond to nonscored items (including added items of assessing history of head trauma in eSAGE that were not included in the original SAGE). Eliminating the time to complete the nonscored items, the median length of time to complete the cognitive testing portion of eSAGE was 14.4 min. Those subjects that took longer to complete the test tended to have lower scores (Spearman correlation; *p* = 0.0001).

#### Sensitivity and specificity for clinical diagnosis

The 66 sample subjects were classified as either dementia (*n* = 21), MCI (*n* = 24), or normal (*n* = 21) based on standard clinical criteria and their neuropsychological testing. ROC of eSAGE based on clinical diagnosis showed an AUC of 0.88; a score of eSAGE at or below 15 provided 71% sensitivity in detecting cognitive impairment (MCI and dementia, *n* = 45) from normal subjects (*n* = 21), and had a specificity of 90%. ROC of SAGE based on clinical diagnosis showed an AUC of 0.83; a score of SAGE at or below 16 provided 69% sensitivity in detecting cognitive impairment from normal subjects, and had a specificity of 86%. There was no statistical difference between eSAGE and SAGE regarding sensitivity (*p* = 0.65), specificity (*p* = 0.56), or AUC (*p* = 0.14). ROC of MoCA based on clinical diagnosis showed an AUC of 0.88; a score of MoCA at or below 23 provided 91% sensitivity in detecting cognitive impairment from normal subjects, and had a specificity of 67%.

We also performed ROC analysis of eSAGE looking at normal vs MCI subjects alone and normal vs dementia subjects alone, resulting in AUC values of 0.78 and 0.99, respectively. A score of 16 or less on eSAGE provided the best combination (in terms of sum) of sensitivity (63%) and specificity (81%) in detecting MCI subjects. Furthermore, a score of 14 or less on eSAGE provided the best combination of sensitivity (95%) and specificity (100%) in detecting dementia. In differentiating nondementia subjects (MCI or normal, *n* = 45) from those with dementia, eSAGE score of 13 or below had a sensitivity of 90% and specificity of 87%; the AUC was 0.92. Likewise, an eSAGE score of 13 or below had a sensitivity of 90% and a specificity of 75% (AUC 0.87) in differentiating MCI subjects from dementia subjects.

#### Clinical diagnosis and cognitive scores

At 5% level of significance, Tukey’s HSD test that performs all pairwise comparisons of the means (Table 3) identified each of the three groups (normal, MCI, and dementia) to be distinct for the 7-item total, eSAGE, SAGE, MMSE, and MoCA. The clinical diagnosis showed highly significant differences between the mean cognitive scores. Both eSAGE and SAGE can distinguish normal from dementia and MCI from dementia (with each *p* < 0.0001). Normal can be distinguished from MCI groups by eSAGE (*p* = 0.004) and SAGE (*p* = 0.04).

**Table 3:** 7-Item total, eSAGE, SAGE, MMSE, and MoCA scores, and their effect sizes

	Normal (*n* = 21)	MCI (*n* = 24)	Dementia (*n* = 21)	Common SD estimate	Effect size d* (normal vs MCI)	Effect size d* (normal vs dementia)
7-Item total	341.4 ± 32.1 (285–403)	280.0 ± 32.1 (223–347)	241.2 ± 33.8 (174–322)	32.70	1.88	3.06
eSAGE (max 22)	18.4 ± 2.1 (15–22)	15.1 ± 3.7 (9–22)	9.1 ± 3.7 (2–17)	3.29	1.00	2.82
SAGE (max 22)	18.9 ± 2.4 (13–22)	16.5 ± 3.3 (11–22)	10.9 ± 3.6 (4–18)	3.17	0.73	2.51
MMSE (max 30)	28.7 ± 1.3 (26–30)	27.3 ± 2.2 (20–30)	24.6 ± 2.2 (21–29)	1.97	0.74	2.10
MoCA (max 30)	24.3 ± 2.9 (19–29)	21.3 ± 2.3 (16–25)	16.3 ± 3.9 (8–23)	3.07	0.99	2.62

Values are shown as mean ± SD (range)

*Cohen’s effect size *d*, defined as the standardized difference between two means, is considered large when *d* ≥0.8. Effect size for comparing normals with cognitively impaired subjects is the average of the last two columns and it is the difference between them for the comparison between MCI and dementia subjects

*eSAGE* electronic Self-Administered Gerocognitive Examination, *max* maximum score, *MCI* Mild Cognitive Impairment, *MMSE* Mini-Mental State Examination, *MoCA* Montreal Cognitive Assessment, *SAGE* Self-Administered Gerocognitive Examination

## Discussion

There are clinical and research advantages to having both validated paper and electronic formats of the same test. The brief office- or bedside-based cognitive tests and the longer traditional neuropsychology batteries are both typically administered with pen and paper. These paper tests have inherent familiarity and understandability for the clinician, clinical researcher, and neuropsychologist, and face validity is high. A validated digital cognitive assessment tool with its equivalent validated paper version brings with it the familiarity of the assessment. It would be very useful to be able to use equivalently scored paper or digital formats to be flexible in having the test be given in the office, in the community, using mobile technology, or on a home tablet. This flexibility is enhanced when both paper and digital tests are self-administered. The practicality of using either paper or electronic versions of a self-administered cognitive assessment tool may increase the number of individuals evaluated for early identification of cognitive impairment, may ease the ability to provide repeated testing to monitor cognitive change over time, and may potentially provide progression prediction.

The digital format has its own set of unique advantages. eSAGE allows the ability to time responses, which may provide enhanced functionality. eSAGE cannot only automatically time how long it takes to answer a question, it can determine how often the individual went back to previous pages, or how often they corrected answers. We plan to evaluate the utility of these metadata in the future. eSAGE will also allow individuals to evaluate themselves online and receive their remotely scored results online to be able to deliver them directly to their healthcare providers. This might make it easier to obtain that baseline cognitive assessment or that follow-up cognitive evaluation to assess for changes in their cognitive abilities in a timely fashion prior to their appointment. Individuals taking eSAGE are instructed to provide their baseline and subsequent test scores to their physician for monitoring progress. Obtaining results of their cognitive status online may be particularly useful to those individuals living in underserved or rural regions with few resources and who do not have easy access to dementia-knowledgeable healthcare providers or advocates. The self-administered digital format may also help reduce the typical stress people experience when taking pen and paper tests in a doctor’s office by a healthcare worker. The digital format also allows providers, researchers, and individuals the ability to store results electronically and thereby avoid having to store or lose paper forms. Digital transfer of information is fast and reliable compared to paper information.

Since the identification of the pre-dementia state of MCI was one of the driving forces behind the development of SAGE, in our study we carefully ensured that a broad range of eSAGE scores in the normal to early dementia range (generally eSAGE scores of 10–22) would be measured against the other neuropsychological tests and against SAGE. The age range and sex distribution of our subjects are typical of the population at risk for MCI and dementia. Our well-educated cohort, typical of individuals willing to participate in studies, does limit what we can conclude about those who are less educated using eSAGE.

Tablet-based eSAGE correlates well with the 7-item total of a battery of neuropsychological tests and performs similarly to the validated SAGE. As would be expected, SAGE and eSAGE scores are highly correlated with each other. SAGE and eSAGE have similar correlation values with MMSE and MoCA. eSAGE has not been compared to other computerized tests. However, based on our results, eSAGE has the qualities to be useful in primary care settings as a brief computerized cognitive assessment tool. It does a fair job in differentiating MCI from both normal and dementia subjects. Its classification accuracy compares well to MoCA with comparable AUC and better specificity but worse sensitivity than MoCA. Effect sizes comparing score means are also similar between eSAGE and MoCA. eSAGE has a practical advantage in the primary care setting over most other computerized and paper tests in that it has both paper and tablet versions.

When translating a written paper test into a digital format given on a tablet device, one cannot assume that the resultant digital cognitive test is identical to the same test administered on paper. There have recently been digital translations of traditional brief pen and paper cognitive tests [57, 76]. It is clear from these attempts that the digital translation needs to be separately validated from the pen and paper version [57]. Factors that can influence differences between computerized cognitive test results and those performed with pen and paper include the individual’s experience and familiarity with digital technology. In addition, administering a test using auditory means by a human (with paper recording) or visually by computer involve separate brain pathways and may lead to different scoring results for the exact same question.

In this study, we are therefore pleased to note that eSAGE not only correlated well but also showed no scale bias compared to SAGE. Since SAGE is self-administered, there are no auditory commands or requests, and only visually read questions and paper responses. eSAGE likewise on the tablet is performed by visually reading the questions, and it does not have aurally provided questions. The main difference between the two tests is the individual’s comfort and experience with the technology and the tablet. It turned out that, in the population tested, on average subjects performed one point worse on eSAGE compared to SAGE. They scored one point less on average when using the digital version of SAGE whether they had normal, mildly impaired, or moderately impaired scores. When we separated out our subjects (47%) who had never had experience with smartphones or tablets, as expected they experienced more difficulties with eSAGE and scored, on average, 1.65 points less whether they had normal, mildly impaired, or moderately impaired scores. Those with experience in using tablet or smartphone devices also scored worse on eSAGE but only by 0.83 points, again without a scale bias. Since an individual’s digital proficiency can be difficult to determine, we suggest adding one point to everyone’s digital scores to get an equivalent paper score. Digital unfamiliarity is likely to fade away over time, as newer generations of individuals will have more exposure and proficiency using digital devices.

When we divided our sample based on clinical diagnosis, we found statistically significant differences between both eSAGE and SAGE mean scores for normal subjects and MCI subjects. We also found statistically significant differences between both eSAGE and SAGE mean scores for MCI subjects and dementia subjects. Cohen’s effect size *d* for eSAGE between the normal and MCI groups (1.0), normal and dementia groups (2.82), normal and cognitively impaired (MCI + dementia) groups (1.91), and MCI and dementia groups (1.82) are all considered large and were slightly higher than those of SAGE. This suggests that eSAGE, like SAGE, does well in differentiating both normal from MCI groups and MCI from dementia groups.

eSAGE is not diagnostic for any specific condition. However, in our sample, eSAGE also had a high level of sensitivity and specificity in distinguishing normal from MCI and mild dementia. This self-administered instrument can be utilized to identify potentially clinically relevant cognitive changes that would then warrant further investigation. What one wants from a case finding tool is a high specificity, and one is more willing to accept false negatives (cognitively impaired that test as normal) rather than risk false positives (normal that test as cognitively impaired). eSAGE combines high specificity with reasonable sensitivity and would work better as a case finding tool than as a screening tool. Over time, if the condition is progressive, the false negatives will convert to true positives and these could be picked up by repeat testing. As might be expected, since subjects performed one point worse on eSAGE compared to SAGE, we found a cutoff score of 16 and above for normal subjects taking eSAGE and 17 and above for normal subjects taking SAGE. Consistently, the best cutoff score for specificity and sensitivity for SAGE from our current sample is the same cutoff value we had in our initial validity study [48]. For eSAGE, in differentiating dementia from nondementia a score of 13 or less for dementia subjects gave the best sensitivity and specificity. Evaluating nondementia subjects alone with eSAGE, a score of 17 or higher for normal subjects provided the best sensitivity and specificity. This suggests that MCI subjects would fall typically in the range of 14–16 on eSAGE. Cutoff total scores are useful as guidelines. Clinicians may gain more clues as to the etiology of cognitive loss by looking at the specific pattern of cognitive deficits in instruments such as eSAGE. Additional helpful information may be obtained from the self-report items in the nonscored part of eSAGE. If the patient scores well on eSAGE, the clinician may determine that no further evaluation is indicated, potentially saving costs for the patient and time for the physician. For patients scoring less well or borderline on eSAGE, the practitioner may wish to continue with a staged screening process such as assessment with an informant screen or further evaluations. We hope eSAGE will allow earlier identification of cognitive impairment so that proper diagnosis and treatment may begin sooner.

### Limitations

Specific limitations related to the SAGE test have been described in previous publications [48, 50].

There are also significant limitations with this study. We extensively studied only 66 subjects, the majority of whom were Caucasian and highly educated. Some caution is needed regarding the interpretation of those with below high school education and also with minorities as they were few in number and not fully represented in our sample. Low-educated subjects have high misclassification rates with other commonly utilized cognitive screening tasks [77]. Results may also be limited based on where the patients were recruited and the range of their cognitive abilities. We attempted to get a broad cross-section of a clinic and a community population. The ADCS-ADL scale, initially designed as an informant-reported measure, was used as a self-report in some subjects when their study partner could not be interviewed. While none of those subjects were believed to have dementia, this could have impacted their ADL scores. In order to test a wide distribution of eSAGE scores, we included eSAGE scores in the normal, MCI, and mild-to-moderate dementia range. Additional longitudinal studies in the future will be very important in evaluating the ability of eSAGE in accurately measuring cognitive change over time. This will help determine if it could help identify conversions from normal to MCI, or MCI to dementia. Further research is also required to determine if eSAGE has utility in identifying early cognitive decline in any specific neurocognitive conditions such as AD, vascular dementia, Parkinson’s disease dementia, dementia with Lewy bodies, frontotemporal dementia, endocrine/metabolic/toxic/oncologic conditions, sleep apnea, or acute confusional states.

Thus far, no large randomized trial has demonstrated a correlation between screening and improved outcomes. This would need to be performed to gain widespread acceptance of screening programs. While this study was primarily looking at correlations between eSAGE and other neuropsychological tests, it is clear that eSAGE will be used primarily by individuals with cognitive concerns or complaints as a way to assess cognition and aid diagnosis. Unless provided by a physician, eSAGE would typically not be taken to obtain a cognitive baseline not in response to a cognitive concern. A self-administered test and a digital test, like eSAGE, would ease the time burden of physicians who desire to incorporate yet another screening evaluation in their clinic setting. The advent of disease-modifying treatments may further justify such screening. Positive screens, however, also impact patients and families who may worry about their future, potential stigma, long-term care, insurance issues, and loss of employment, driving, and independence. A staged screening approach reducing the number of false-positive screens would improve the comfort level of physicians and patients with cognitive screening programs.

## Conclusions

eSAGE performed similarly with the 7-item total of a battery of neuropsychological tests, MoCA, and MMSE, and shows no scale bias compared to the validated SAGE. The slope of SAGE versus eSAGE is not significantly different from 1 whether or not the subject was digitally proficient, showing strong evidence that the scaling is identical between eSAGE and SAGE (no scale bias). There is a slight decrease in the eSAGE score when compared to the SAGE score, and the decrease is under a point for digitally proficient subjects. SAGE and eSAGE scores are highly correlated with each other (0.88) and have similar correlation values with MMSE and MoCA. This study also suggests that eSAGE and SAGE have a high level of sensitivity and specificity in distinguishing normal from MCI and mild dementia. eSAGE has the advantage of self-administration, brevity, four interchangeable forms, and potential widespread availability to be a major factor in overcoming the many obstacles in identifying early cognitive changes in individuals.

## Abbreviations

AD: Alzheimer’s disease
ADL: Activities of daily living
AUC: Area under the curve
eSAGE: Electronic Self-Administered Gerocognitive Examination
HVLT: Hopkins Verbal Learning Test
MCI: Mild cognitive impairment
MMSE: Mini-Mental State Examination
MoCA: Montreal Cognitive Assessment
NPI: Neuropsychiatric Inventory
ROC: Receiver operating characteristics
SAGE: Self-Administered Gerocognitive Examination
WAIS III: Wechsler Adult Intelligence Scale III
WCST: Wisconsin Card Sort Test
